# Editorial: Integrated Point-of-Care Testing (POCT) Systems: Recent Progress and Applications

**DOI:** 10.3389/fbioe.2022.851675

**Published:** 2022-03-01

**Authors:** Lirong Wang, Tailin Xu

**Affiliations:** ^1^ School of Biomedical Engineering, Health Science Center, Shenzhen University, Shenzhen, China; ^2^ Research Center for Bioengineering and Sensing Technology, University of Science and Technology Beijing, Beijing, China

**Keywords:** wearables, POCT, flexible biomaterials, bioelectronics, biosensors, portable devices, disease diagnostics, healthy monitoring

Traditional diagnostic methods for various biomarkers and biochemical parameters are normally carried out in specialized laboratories with professional workers, which leads to long assay times, high reagent consuming, and low efficiency (Shrivastava et al., 2020). Nowadays, point-of-care testing (POCT) technology with miniaturization of diagnostic tools has been successfully commercialized for health monitoring and disease diagnostics (Kim et al., 2019; Yang and Gao, 2019). Compared with traditional laboratory testing, POCT technology exhibits the attractive properties of small batches of samples, less reagent usage, user-friendliness, easy-to-use format, compact appearance, and fast turnaround time (Ray et al., 2019; Liu et al., 2020; Qiao et al., 2020). Recently, with emerging technological innovations in chemical and biochemical sensors, bioelectronics, and wearable electronics, the development of integrated POCT systems has an unprecedented opportunity to achieve personal healthcare and precision medicine (Gao et al., 2016; Luo et al., 2020; Ates et al., 2021; Sempionatto et al., 2021; Wang et al., 2021).

In this special topic issue, 14 papers have been included, with a mixture of 10 original research articles, 4 reviews. This research topic aims at discussing recent progress and applications of integrated POCT systems, including user-friendly, portable and simple monitoring platforms; immediate clinical assessment technology; flexible wearable sensors based on nanomaterials; and highly sensitive and specific biosensors. In addition, these integrated POCT systems are also widely applied in clinical diagnosis, disease control and other fields through the detection of various biomarkers.

For example, large-scale rapid POCT systems for infectious diseases are urgently needed during the COVID-19 pandemic. Wang et al. reported a novel diagnostic testing platform based on the principle of real-time reverse transcription loop-mediated isothermal amplification for the diagnosis of COVID-19, which can complete the entire testing process from sample Research Topic to result interpretation within 55 min. In addition, research progress in developing paper-based POC devices for SARS-CoV-2 detection was reviewed by Jia et al., including the detection principle, analysis performance, advantages and disadvantages of existing methods and equipment.

Recently, wearable sensors capable of continuously monitoring human physiological signals in real-time have attracted a lot of research interest with the emergence of flexible bioelectronics and biomaterials. Therefore, Cheng et al. comprehensively reviewed the research progress of intelligent wearable sensors for detecting various biological fluids, including sweat, blood, interstitial fluid, tears and wound fluid, and made concluding comments to guide their better development in commercialization and practicability. Yan et al. summarized the application of flexible biosensors in POCT and classified them according to substrate materials (polymers, paper, and textiles) to help readers gain a comprehensive understanding of the development trends in the field. Moreover, Raza et al. also introduced the research progress of wearable electrochemical biosensors to readers from the perspective of conductive nanomaterials. Furthermore, a flexible sensor for simultaneous detection of ascorbic acid, dopamine, and uric acid is fabricated by growing 3D graphene nanosheets on flexible carbon cloth through thermal chemical vapor deposition, with linear monitoring ranges of 0.02–0.1, 0.0005–0.02, and 0.0005–0.02 mM, respectively, (Meng et al.). Fu et al. proposed a strategy that can not only effectively inhibit bacterial adhesion (the antibacterial efficiency remains above 66%), but also capture and detect nickel ions through chelation reaction (the capture efficiency is about 57% at 5 days), which can be used in implantable biomedical materials and equipment in the future.

More importantly, the combination of POCT with nano-diagnostics technology has become the latest trend in the development of fully integrated POCT systems in the field of personal healthcare. A novel approach for the visual detection of the Hepatitis B virus in the clinical application was first developed by Chen et al. by integrating loop-mediated isothermal amplification with a gold nanoparticle-based lateral flow biosensor, and the entire detection process includes genomic DNA extraction (∼10 min), preamplification (∼40 min), and LFB readout (∼2 min) can be completed in 60 min. Similarly, Wang et al. (2021) developed a novel detection method called “CRISPR-HBV” for Hepatitis B virus diagnosis, which can detect 10 copies of genomic DNA with 100% specificity. He et al. provide a promising point-of-care biosensor for a biomarker of Alzheimer’s disease (Amyloid-β protein) in which ZnO@polydopamine/Au nanocomposites has excellent visible-light activity, possessing a wide Linear range (1 pg/ml to 100 ng/ml) and low detection limit (0.26 pg/ml). An intelligent multi-modal point-of-care system combining clinical indicators with CT images was developed to predict transarterial chemoembolization response in hepatocellular carcinoma with 98% accuracy (Sun et al.). A point-of-care testing system based on a magnetically propelled microdimer was reported for the quantification analysis of plasma glucose, cholesterol, and triglyceride concentrations (Yu et al.). Hwang et al. presented a magnetic force-assisted electrochemical sandwich immunoassay for the quantitative analysis of carcinoembryonic antigen, which showed a linear relationship of 0.5–200 ng/ml and a detection limit of 0.50 ng/ml. Sun et al. developed a method for the timely diagnosis of B. pertussis infections that combines loop-mediated isothermal amplification and a nanoparticle-based lateral flow biosensor with a sensitivity of 50 fg per reaction. Notably, these works require further follow-up and provide more clinical data to confirm their practicability and accuracy.

In conclusion, we have included recent advances and applications of integrated point-of-care testing (POCT) systems in this topic. We hope to bring readers a timely and interesting overview to inspire future attention to multidisciplinary research in materials science, chemistry and biotechnology to address the remaining challenges of integrated POCT systems for personal healthcare. For example, a powerful wearable sensing system integrating new materials, sensing technology and a seamless system is lacking, which can accurately and continuously collect and output data from the human body. Miniaturization and integration of signal processing systems need to be explored to fully exploit the potential of flexible electronics. Considering the complexity of the human body, future research work should also focus on human comfort studies, especially friendly sampling methods and flexible systems that mechanically match the skin interface. Further research in these directions could accelerate the commercialization of such integrated POCT systems, which will have a significant impact on personalized healthcare.
